# Dietary food patterns and glucose/insulin homeostasis: a cross-sectional study involving 24,182 adult Americans

**DOI:** 10.1186/s12944-017-0571-x

**Published:** 2017-10-04

**Authors:** Mohsen Mazidi, Andre Pascal Kengne, Dimitri P. Mikhailidis, Peter P. Toth, Kausik K. Ray, Maciej Banach

**Affiliations:** 1 Key State Laboratory of Molecular Developmental Biology, Institute of Genetics and Developmental Biology, Chinese Academy of Sciences, Chaoyang, Beijing, China; 2 Institute of Genetics and Developmental Biology, College, University of Chinese Academy of Science, Beijing, China; 3 Non-Communicable Disease Research Unit, South African Medical Research Council and University of Cape Town, Cape Town, South Africa; 4 Department of Clinical Biochemistry, University College London Medical School, University College London (UCL), London, UK; 5 Department of Preventive Cardiology, CGH Medical Center, Sterling, IL USA; 6 Ciccarone Center for the Prevention of Cardiovascular Disease, Johns Hopkins University School of Medicine, Baltimore, MD USA; 7 Department of Public Health and Primary Care, University of Cambridge, Cambridge, UK; 8 Department of Hypertension, Chair of Nephrology and Hypertension, Medical University of Lodz, Lodz, Poland; 9 Polish Mother’s Memorial Hospital Research Institute (PMMHRI), Lodz, Poland; 10 Cardiovascular Research Centre, University of Zielona-Gora, Zielona-Gora, Poland

**Keywords:** Dietary patterns, Insulin resistance, Glucose homeostasis, Insulin homeostasis

## Abstract

**Aim:**

To investigate the association of major dietary patterns with glucose and insulin homeostasis parameters in a large American sample. The association between dietary patterns (DP) derived via principal components analysis (PCA), with glucose/insulin homeostasis parameters was assessed. The likelihood of insulin resistance (IR) across the DPs quarters was also explored.

**Method:**

The United States National Health and Nutrition Examination Survey (NHANES) participants during 2005–2012 were included if they underwent measurement of dietary intake as well as glucose and insulin homeostasis parameters. Analysis of covariance (ANCOVA) and adjusted logistic and linear regression models were employed to account for the complex survey design and sample weights.

**Results:**

A total of 24,182 participants were included; 48.9% (*n* = 11,815) were men. Applying PCA revealed three DP (56.8% of variance): the first was comprised mainly of saturated fat (SFA), total fat, mono-unsaturated fatty acids (MUFA) and carbohydrate (CHO); the second is highly enriched with vitamins, trace elements and dietary fiber; and the third was composed of polyunsaturated fatty acids (PUFA), cholesterol and protein. Among the total population, after adjustment for age, sex, race, C-reactive protein, smoking, and physical activity, glucose homeostasis factors, visceral adiposity index and lipid accumulation product improved across the quarters of the first and third DP; and a reverse pattern with the second DP. The same trend was observed for the non-diabetic subjects. Moreover, subjects with higher adherence to the first and third DP had higher likelihood for developing IR, whereas there was a lower likelihood for the second DP.

**Conclusion:**

This study shows that the DP heavily loaded with CHO, SFA, PUFA, protein, total fat and MUFA as well as high-cholesterol-load foods is associated with impaired glucose tolerance; in contrast, the healthy pattern which is high in vitamins, minerals and fiber may have favourable effects on insulin sensitivity and glucose tolerance.

## Background

β-Cell dysfunction and hyperinsulinaemia are involved in the development of diabetes mellitus (DM) [1–3]. The triglycerides/glucose index (TyG), a product of triglycerides (TG) and fasting blood glucose (FBG), has also been used as an alternative marker of IR among adults [4, 5]. Recent investigation has reported that the TyG index could be a better alternative marker of IR among adolescents compared with the homeostatic model assessment-insulin resistance (HOMA-IR) [5]. There are complex interactions between social, behavioural, cultural, physiological, metabolic and genetic factors that are involved in the development of dysglycaemia and hyperinsulinaemia. It is widely accepted that dietary factors are important in preventing impaired glucose metabolism [6, 7]. Relatively limited nutritional epidemiological research has been conducted to examine long-term effects of diet on glucose and insulin homeostasis, and previous studies have focused mainly on the risk of DM.

Studies on the impact of diet on health have traditionally focused on single nutrients or food/food groups. These approaches, however, have methodological and conceptual limitations [8–10], in the sense that they can only capture the effects of a single nutrient or food group, but not the interactions among nutrients and foods [10, 11]. Dietary pattern (DP) analysis is an alternative approach in nutritional epidemiology [8, 12, 13]. In this approach, statistical methods are used to examine the pattern of intake of multiple foods or nutrients and derive single-exposure variables, or DPs [9]. Using the DP approach could facilitate the development of public health recommendations that are more convenient to follow [14].

Although the relationship between glucose tolerance abnormalities and dietary factors in terms of dietary intake and food patterns has been investigated, the conclusions are still contradictory [15–17]. These findings may be explained by various methodologies, such as the different techniques used for measuring diet, food patterns, or blood glucose; study design; or study population. The aim of the current study was to examine the association between DP and glucose and insulin homeostasis in a cross section of American adults.

## Methods

### Population

Data for participants who took part in the Nutrition and Health Examination Surveys (NHANES) from 2005 to 2012 were used [18], and were restricted to those aged ≥18 years. NHANES, which has been extensively described, is an ongoing cycle of cross-sectional surveys conducted by the US National Centre for Health Statistics (NCHS) [18]. Briefly, in NHANES, complex, multistage, probability sampling procedures are used to ensure selection of participants from various geographical locations and racial/ethnic populations [18, 19]. Trained interviewers collected participants’ demographic, socioeconomic, dietary, and health-related information using questionnaires administered during home visits, whereas clinical examination and dietary assessment are conducted by skilled personnel using a mobile examination centre (MEC) [18]. Further details on protocols for biochemical analyses are available elsewhere; all methods were carried out in accordance with relevant guidelines and regulations [18]. All experimental protocols were approved by the National Centre for Health Statistics [18]. Informed consent was obtained from all adult participants, and the National Centre for Health Statistics Research Ethics Review Board approved the protocol. A blood specimen was drawn from the participant’s antecubital vein by a trained phlebotomist according to a standardized protocol. Fasting glucose was measured in plasma by a hexokinase method using a Roche/Hitachi 911 Analyser and Roche Modular P Chemistry Analyser. Details on high sensitivity C-reactive protein (hsCRP) concentrations measurement are available elsewhere [20]. Insulin was measured using an ELISA immunoassay (Mercodia, Uppsala, Sweden) [21]. Further details on protocols for biochemical analyses are available elsewhere [20–23].

Homeostatic model assessment of insulin resistance (HOMA-IR), β-cell function (HOMA-B) and insulin sensitivity (HOMA-IS) were calculated as follows: the homeostatic model of insulin resistance (HOMA-IR) = [glucose (nmol/L) * insulin (mU/mL)/22.5] using fasting values, HOMA-B = [20 × fasting insulin (μU/ml)]/ [fasting glucose (mmol/l) − 3·5], and HOMA-S = 1/HOMA-IR× 100 [24]. These indices have been developed as validated alternative tools for assessment of insulin homeostasis in epidemiological studies. The triglyceride-glucose (TyG) index was calculated as the ln[fasting triglyceride(TG) (mg/dl) × glucose (mg/dl)/2] [25]. DM was diagnosed by self-reported history of DM or fasting plasma glucose (FBG) ≥126 mg/dl [26]; HOMA-IR >2.5 indicates insulin resistance [27]. The anthropometrically predicted visceral adipose tissue (apVAT) was predicted with sex-specific validated equations that included age, BMI, and circumferences of the waist and thigh [28]. The formula for men was: 6 *waist circumference – 4.41 * proximal thigh circumference + 1.19 * age – 213.65; and the formula for women was: 2.15 * waist circumference – 3.63 * proximal thigh +1.46 * age + 6:22 * BMI -92.713 [28]. Visceral adiposity index (VAI) was calculated using sex-specific formulas: males [WC/39.68 + (1.88 × BMI)] × (TGs/1.03) × (1.31/HDL); Females: [WC/36.58 + (1.89 × BMI)] × (TGs/0.81) × (1.52/HDL), where both TGs and high density lipoprotein (HDL) levels are expressed in mmol/L [29]. Lipid accumulation product (LAP) index, a novel measure of central lipid accumulation and predictor of metabolic syndrome and cardiovascular disease, was calculated as [WC (cm)–65] × [triglycerides (mmol/L)] in men, and [WC (cm)–58] × [triglycerides (mmol/L)] in women [30]. Smoking status was self-reported.

### Statistical analysis

Statistical analyses used the complex sample module of the SPSS® software version 22.0 (IBM Corp, Armonk, NY), applying the Centers for Disease Control (CDC) guidelines for complex NHANES data analysis, accounting for the masked variance and recommended weighting scheme [31]. The principal component analysis (PCA) was applied to determine the dietary patterns as previously described [32]. Briefly, orthogonal transformation (varimax procedure) was applied to derive nutrient patterns based on the nutrient compounds. Factors were retained for further analysis based on their natural interpretation and eigenvalues on the Scree test [32, 33]. Factor score for each dietary pattern was calculated by summing intakes of nutrients weighted by their factor loadings [32, 33]. Adjusted (age-, sex-, race-, C-reactive protein, smoking, physical activity) mean of glucose and insulin homeostasis factors across the quarters of each food pattern, was calculated. Adjusted (age-, sex-, race-, C-reactive protein, smoking, physical activity) logistic regressions were applied to determine risk of insulin resistance across the quarters of each dietary pattern. A two-sided *p* < 0.05 was used to identify statistically significant results.

## Results

A total of 24,182 participants met the criteria for inclusion in the current analyses. Their characteristics are summarised in Table 1; 11,815 (48.9%) participants were men and 12,367 (51.1%) were women; their mean age was 45.9 years. Non-Hispanic white (68.5%) was the largest racial group and other Hispanic (5.0%) the smallest racial group. Furthermore, 50.2% of the participants were married, while 28.8% had achieved more education than high school (Table 1). As seen from Table 1, mean and standard error mean (SEM) of FBG and plasma insulin were 98.27 ± 0.12 (mg/dl) and 12.92 ± 0.15 (μU/mL), respectively. Moreover, when participants were divided based on their DM status, it was found that in the DM group, men were the majority (53.1%) while in the non-DM group women (51.6%) were more common (*p* < 0.001). DM subjects were older than the non-DM subjects (59.6 vs. 46.1 years). DM subjects had significant higher level of LAP, VAI, TyG index, Serum TG/HDL ratio and apVAT (all p < 0.001).

**Table 1:** Demographic characteristics of subjects

Characteristics		Overall	Non-diabetic subjects	Diabetic subjects	*p*-value
Sex	Men (%)	48.9	48.4	53.1	<0.001
	Women (%)	51.1	51.6	46.9	
Age (Years)		45.9 ± 0.2	46.1 ± 0.2	59.6 ± 0.3	<0.001
Race/Ethnicity	White (non-Hispanic) (%)	68.5	48.3	42.3	<0.001
	Non-Hispanic Black (%)	11.6	19.7	21.4	
	Mexican-American (%)	8.3	18.9	22.7	
	Other Hispanic (%)	5.0	8.5	8.5	
	Other (%)	6.6	4.6	5.0	
Marital Status	Married (%)	50.2	51.4	55.4	<0.001
	Widowed (%)	8.8	7.5	15.1	
	Divorced (%)	10.2	9.9	12.3	
	Never married (%)	18.9	19.7	9.2	
Education Status	Less than high school (%)	12.2	11.7	21.4	<0.001
	Completed high school (%)	39.0	39.8	41.8	
	More than high school (%)	48.8	48.5	36.7	
Body mass index (kg/m^2^)		28.5 ± 0.1	28.4 ± 0.1	31.8 ± 0.2	<0.001
Waist circumference (cm)		98 ± 0.3	97 ± 1	107 ± 1	<0.001
Anthropometrically predicted visceral adipose tissue		179.9 ± 1.8	174.8 ± 2.3	249.2 ± 1.9	<0.001
Serum triglycerides (mg/dl)		154 ± 3	147 ± 3	215 ± 4	<0.001
Serum total cholesterol (mg/dl)		196 ± 1	190 ± 3	195 ± 2	<0.001
Serum high density lipoprotein (mg/dl)		53 ± 1.6	53 ± 2	47.1 ± 1	<0.001
Serum TG/HDL ratio		3.5 ± 0.1	3.3 ± 0.1	5.5 ± 0.7	<0.001
Serum hsCRP (mg/dl)		0.39 ± 0.05	0.40 ± 0.06	0.63 ± 0.02	<0.001
Serum Apolipoprotein (B) (mg/dL)		93 ± 2	95 ± 3	97 ± 2	<0.001
Systolic blood pressure (mmHg)		121 ± 1	122 ± 0.3	132 ± 1	<0.001
Diastolic blood pressure (mmHg)		70 ± 1	68 ± 0.4	70 ± 0.3	<0.001
Fasting blood glucose (mg/dl)		98 ± 0.1	91 ± 0.4	182 ± 1	<0.001
Plasma Insulin (μU/mL)		12.9 ± 0.1	12.7 ± 0.4	22.3 ± 0.1	<0.001
HOMA-IR		3.3 ± 0.08	2.9 ± 0.08	9.6 ± 0.06	<0.001
HOMA-B		15.6 ± 2.5	162.4 ± 3.6	85.5 ± 1.2	<0.001
HOMA-S		0.56 ± 0.01	0.60 ± 0.02	0.25 ± 0.01	<0.001
HbA1c (%)		5.5 ± 0.01	5.4 ± 0.01	7.4 ± 0.02	<0.001
2-h blood glucose (mg/dL)		116 ± 1	117 ± 1	273 ± 0.3	<0.001
TyG index		8226 ± 72	6785 ± 83	20,590 ± 234	<0.001
Lipid Accumulation Product		68 ± 0.5	63 ± 0.2	112 ± 0.9	<0.001
Visceral Adiposity Index		2.5 ± 0.02	2.3 ± 0.03	4.0 ± 0.05	<0.001

Value are expressed as a mean and standard error of the mean (SEM) or percentage. HOMA-IR, Homeostatic model assessment of insulin resistance; HOMA-B, Homeostatic model assessment of β-cell function HOMA-S; Homeostatic model assessment of insulin sensitivity TyG index, triglyceride-glucose index hsCRP; high sensitivity C-reactive protein, HbA_1c_ glycated haemoglobin, TG/HDL ratio; triglyceride to high density lipoprotein. *P* values are for the comparison between “Non-diabetic “and “Diabetic “subjects

Applying PCA resulted in three dietary patterns which together explained 56.8% of variance in dietary nutrient consumption. The first dietary pattern (22.6%) consisted of saturated fat (SFA), total fat, mono-unsaturated fatty acids (MUFA) and carbohydrate; the second dietary pattern (18.9%) was enriched with vitamins, trace elements and dietary fiber; and the third dietary pattern (15.3%) was enriched with polyunsaturated fatty acids (PUFA), cholesterol and protein.

The age-, sex-, race-, hsCRP, smoking, and physical activity adjusted mean of glucose homeostatic factors across the quarters of dietary patterns are shown in Table 2. Across quarters of the first dietary pattern, mean levels of markers of glucose and insulin homeostasis increased for plasma insulin (12.3 vs 15.5 μU/mL), HbA1c (5.6 vs. 5.8%), HOMA-IR (3.3 vs.4.1), HOMA-B (143.5 vs. 164.7), VAI (2.2 vs. 2.7), LAP (63.2 vs.73.8) and decreased for HOMA-S (0.58 vs. 0.49) (all *p* < 0.001). The pattern was reversed across quarters of the second dietary pattern, as FBG (102.2 vs. 99.1 mg/dl), plasma insulin (14.5 vs. 12.7 μU/mL), HbA1c (5.8 vs. 5.4%), 2 h–glucose (123.1 vs.115.4 mg/dl), HOMA-IR (3.9 vs. 3.3), VAI (2.6 vs. 2.1), LAP (70.6 vs. 62.8) decreased while HOMA-S (0.50 vs. 0.58) increased (all p < 0.001). The profiles of indicators of glucose and insulin homeostasis across quarters of the third DP were similar to those across quarters of the first DP, although changes in HOMA-B, HOMA-S and VAI were not significant (Table 2). When analysis was restricted to participants without known DM (91.4% of the total sample), the results remained similar to those observed in the total sample, with only few exceptions. These exceptions related to mean HbA_1c_ across quarters of the first and third DP, and fasting glucose across quarters of the third DP, which were no longer significant (Table 2).

**Table 2:** Adjusted (age-, sex-, race-, C-reactive protein, smoking, physical activity) mean of glucose homeostasis factors across the quarters of the dietary patterns

Character		First Dietary Pattern					Second Dietary Pattern					Third Dietary Pattern				
		Q1	Q2	Q3	Q4	*P*-value	Q1	Q2	Q3	Q4	*P*-value	Q1	Q2	Q3	Q4	*P*-value
total	Fasting blood glucose (mg/dl)	100 ± 1	101.6 ± 0.4	101.2 ± 0.5	102.5 ± 0.3	0.123	102.2 ± 0.1	101.8 ± 0.5	100.2 ± 0.5	99.1 ± 0.7	<0.001	100.5 ± 0.3	100.6 ± 0.4	101.1 ± 0.3	102.4 ± 0.4	<0.001
	Plasma Insulin (μU/mL)	12.3 ± 0.3	13.5 ± 0.5	14.6 ± 0.4	15.5 ± 0.2	<0.001	14.5 ± 0.3	14.0 ± 0.2	13.2 ± 0.4	12.7 ± 0.6	<0.001	12.1 ± 0.3	13.2 ± 0.7	14.0 ± 0.3	14.2 ± 0.2	<0.001
	HbA1c (%)	5.6 ± 0.01	5.7 ± 0.01	5.7 ± 0.03	5.8 ± 0.06	<0.001	5.8 ± 0.01	5.7 ± 0.01	5.6 ± 0.01	5.4 ± 0.02	<0.001	5.6 ± 0.03	5.6 ± 0.04	5.7 ± 0.04	5.7 ± 0.05	<0.001
	2 h–Glu (mg/dl)	121.5 ± 1.4	118.8 ± 1.2	121.4 ± 1.7	122.8 ± 1.6	0.526	123.1 ± 1.2	122.5 ± 1.3	119.2 ± 1.7	115.4 ± 1.9	<0.001	120.4 ± 1.4	119.1 ± 1.7	119.5 ± 1.1	120.0 ± 1.2	0.109
	HOMA-IR	3.3 ± 0.1	3.5.9 ± 0.1	3.8 ± 0.1	4.1 ± 0.1	<0.001	3.9 ± 0.2	3.7 ± 0.1	3.6 ± 0.1	3.3 ± 0.1	<0.001	3.4 ± 0.1	3.4 ± 0.1	3.7 ± 0.1	3.9 ± 0.1	<0.001
	HOMA-B	143.5 ± 5.1	144.5 ± 2.6	160.2 ± 6.5	164.7 ± 4.2	<0.001	150 ± 2.6	153.1 ± 3.8	158.7 ± 4.1	150.4 ± 2.9	0.415	147.6 ± 4.1	148.0 ± 8.2	163.1 ± 6.3	151.1 ± 4.1	0.225
	HOMA-S	0.58 ± 0.01	0.55 ± 0.06	0.46 ± 0.01	0.49 ± 0.02	<0.001	0.50 ± 0.01	0.51 ± 0.09	0.54 ± 0.03	0.58 ± 0.04	<0.001	0.54 ± 0.05	0.53 ± 0.04	0.52 ± 0.01	0.54 ± 0.02	0.125
	TyG index	8199.9 ± 180.2	8578.7 ± 213.6	8716.8 ± 215.2	8835.8 ± 231.4	0.415	8415.4 ± 232.3	8718.4 ± 192.6	8753.6 ± 178.2	8356.3 ± 192.3	0.102	8365.2 ± 235.1	8424.8 ± 241.3	8611.0 ± 202.2	8833.9 ± 210.3	<0.001
	Visceral Adiposity Index	2.2 ± 0.02	2.4 ± 0.03	2.5 ± 0.07	2.7 ± 0.06	<0.001	2.6 ± 0.05	2.5 ± 0.01	2.4 ± 0.06	2.1 ± 0.09	<0.001	2.6 ± 0.04	2.5 ± 0.06	2.6 ± 0.01	2.6 ± 0.08	0.324
	Lipid Accumulation Product	63.2 ± 1.5	66.3 ± 2.0	70.5 ± 1.9	73.8 ± 1.6	<0.001	70.6 ± 1.7	69.4 ± 2.0	68.6 ± 1.3	62.8 ± 2.8	<0.001	64.4 ± 1.4	65.6 ± 1.0	69.1 ± 1.6	70.0 ± 1.3	<0.001
Without DM	Fasting blood glucose (mg/dl)	91.3 ± 0.3	91.6 ± 0.4	92.4 ± 0.6	91.1 ± 0.4	0.325	92.9 ± 0.3	91.1 ± 0.2	91.2 ± 0.4	90.8 ± 0.3	<0.001	90.1 ± 0.7	91.7 ± 0.4	91.8 ± 0.2	91.1 ± 0.3	0.724
	Plasma Insulin (μU/mL)	12.1 ± 0.2	12.3 ± 0.1	13.0 ± 0.09	14.0 ± 0.1	<0.001	13.5 ± 0.1	13.0 ± 0.1	12.6 ± 0.1	12.4 ± 0.1	<0.001	12.1 ± 0.1	12.9 ± 0.1	13.1 ± 0.1	13.6 ± 0.0	<0.001
	HbA1c (%)	5.4 ± 0.01	5.4 ± 0.01	5.4 ± 0.02	5.4 ± 0.03	0.725	5.5 ± 0.02	5.5 ± 0.01	5.4 ± 003	5.3 ± 0.01	<0.001	5.4 ± 0.01	5.4 ± 0.02	5.4 ± 0.01	5.4 ± 0.01	0.415
	2 h–Glu (mg/dl)	117.1 ± 0.1	113.1 ± 1.2	113.3 ± 1.2	114.5 ± 1.3	0.623	116.4 ± 1.7	115.8 ± 1.4	113.7 ± 1.2	111.1 ± 1.4	<0.001	114.4 ± 2.0	113.6 ± 1.3	115.4 ± 2.0	114.2 ± 1.8	0.328
	HOMA-IR	2.8 ± 0.1	2.9 ± 0.1	3.0 ± 0.2	3.3 ± 0.1	<0.001	3.1 ± 0.1	3.0 ± 0.1	2.9 ± 0.1	2.6 ± 0.1	<0.001	2.6 ± 0.1	2.7 ± 0.1	3.1 ± 0.09	3.2 ± 0.1	<0.001
	HOMA-B	146.6 ± 2.6	148.9 ± 3.2	167.0 ± 5.6	173.0 ± 3.4	<0.001	155.9 ± 2.8	158.4 ± 3.4	150.2 ± 2.6	155.7 ± 4.6	0.415	152.8 ± 2.3	151.6 ± 4.1	170.1 ± 3.2	151.0 ± 2.1	0.462
	HOMA-S	0.56 ± 0.01	0.59 ± 0.02	0.56 ± 0.06	0.54 ± 0.01	<0.001	0.50 ± 0.01	0.53 ± 0.01	0.60 ± 0.02	0.62 ± 0.04	<0.001	0.55 ± 0.03	0.58 ± 0.01	0.55 ± 0.01	0.60 ± 0.02	0.328
	TyG index	6592.3 ± 123.5	6970.9 ± 182.3	7189.9 ± 142.6	7289.8 ± 172.3	<0.001	7025.6 ± 145.9	6937.6 ± 122.5	7156.9 ± 147.6	6856.4 ± 176.3	0.109	6974.3 ± 134.5	6979.2 ± 138.6	7060.8 ± 174.3	6961.2 ± 162.9	0.715
	Lipid Accumulation Product	2.2 ± 0.08	2.4 ± 0.04	2.5 ± 0.06	2.6 ± 0.01	<0.001	2.2 ± 0.05	2.1 ± 0.09	2.0 ± 0.04	1.8 ± 0.06	<0.001	2.4 ± 0.04	2.4 ± 0.06	2.3 ± 0.04	2.4 ± 0.08	0.136
	Visceral Adiposity Index	57.0 ± 2.3	61.2 ± 1.0	67.5 ± 2.0	69.4 ± 1.4	<0.001	65.6 ± 2.8	62.4 ± 1.4	61.6 ± 2.0	58.4 ± 1.9	<0.001	60.4 ± 1.8	61.1 ± 1.4	64.1 ± 2.0	65.5 ± 1.7	<0.001

Analysis of covariance conducted to estimate the adjusted means. Value are expressed as a mean and standard error of the mean (SEM) or percentage. HOMA-IR, Homeostatic model assessment of insulin resistance; HOMA-B, Homeostatic model assessment of β-cell function HOMA-S; Homeostatic model assessment of insulin sensitivity, TyG index, triglyceride-glucose index hsCRP; high sensitivity C-reactive protein, HbA_1c_ glycated haemoglobin

In age-, sex-, race-, hsCRP, smoking, and physical activity adjusted logistic regression, the likelihood of insulin resistance increased across quarters of the first and third DP, but decreased across quarters of the second DP. For example, relative to the lowest quarters, the odds ratio (95% confidence interval) for prevalent insulin resistance was 1.14 (1.00–1.31, *p* < 0.001) for the second quarter, 1.37 (1.14–1.63, p < 0.001) for the third quarter and 1.50 (1.26–1.79, p < 0.001) for the fourth quarter of the first DP. Equivalent figures were 0.99 (0.84–1.16, *p* = 0.223), 0.88 (0.76–1.01, *p* = 0.125) and 0.69 (0.60–0.80, p < 0.001) across quarters of the second DP, and 1.03 (0.89–1.20, *p* = 0.423), 1.25 (1.08–1.45, p < 0.001) and 1.33 (1.18–1.51, p < 0.001) across quarters of the third DP.

## Discussion

The results of this study reveal that subjects who consumed higher fat, PUFA, protein, carbohydrate and cholesterol had less favourable profiles of glucose and insulin homeostasis, while subjects with higher intake of vitamins, trace elements and dietary fiber had favourable profiles.

In accordance with findings from the current study, other investigators have reported that a diet rich in fruits, vegetables, and whole grains prevents or controls insulin resistance related states including impaired fasting glucose (IFG) and impaired glucose tolerance (IGT) [6]; However, consistent consumption of refined grains, high-fat dairy and sugar sweetened beverages, and a diet rich in saturated fatty acids and cholesterol increases the risk of impaired glucose and insulin regulation [34].

An association between green leafy vegetables (which are enriched with vitamins, trace elements and soluble dietary fiber) with a reduced risk of type 2 diabetes has also been reported [35, 17]. Furthermore, the Nurses’ Health Study showed that a dietary pattern high in refined carbohydrate and sugar such as sugar-sweetened soft drinks, refined grains, soft drinks and processed meat, but low in cruciferous vegetables and yellow vegetables, correlated with augmented risk for type 2 DM [36]. The Mediterranean or DASH (Dietary Approaches to Stop Hypertension) diets exert a protective effect against development of insulin resistance and type 2 DM [37]. This supports the notion that a plant-based food pattern with a balanced glycaemic index and load, rich in soluble fiber and phytochemicals, would be effective to reduce risk of dysglycaemia and prediabetes states. The Mediterranean and DASH diets are relatively rich in fat from vegetable sources (extra-virgin olive oil, tree nuts) and include an abundance of minimally processed plant-foods (vegetables, fruits, whole grains, legumes), moderate fish consumption, low consumption of meat and meat products, and wine in moderation, usually consumed with meals [38]. It has been proposed that their favourable impact could be due to their components [38]. For example, in meta-analysis and systematic review of 17 original research studies (1 clinical trial, 9 prospective and 7 cross-sectional) of 136,846 participants, higher adherence to the Mediterranean diet was associated with a 23% reduced risk of developing type 2 diabetes [39]. There is a plausible biological explanation. The antioxidant profile of the diet may suppress oxidative stress accumulation, which has been reported to mediate the development of insulin resistance and β-cell dysfunction [40]. In addition, magnesium-rich foods, such as vegetables, nuts and legumes can prevent a magnesium deficiency. It was found that decreased intracellular enzymatic activity, attributed to magnesium deficiency, might favour insulin resistance [41], while extracellular magnesium is also needed to prevent a rise in intracellular calcium concentration, which impairs insulin signalling as well [42]. Another mechanism implicates soluble dietary fiber, particularly those found in cereal [43]. Their beneficial properties could derive from high magnesium concentrations or delayed gastric emptying, which slows down digestion and glucose absorption and reduces plasma insulin levels [44]. Moreover, moderate alcohol consumption has been associated with enhanced insulin sensitivity, possibly through adiponectin or HDL-C [45], whereas resveratrol, a phytophenol primarily found in wine, has also been implicated in improved insulin signalling [45].

Consistent with current findings, in subjects without DM, the Framingham Offspring Study showed that adherence to both the ‘refined grains and sweets’ and ‘soda’ dietary patterns compared with the ‘dietary pattern high in fruits or reduced fat dairy and whole grains’ pattern (Mediterranean diet) were associated with higher 2 h post-challenge insulin levels [46, 47]. Moreover, confectionaries and soft drinks, may deteriorate the glucose metabolism according to the possibly unfavourable effect of diets high in simple sugars on insulin sensitivity [48].

This study has several strengths. The large sample provided adequate statistical power to evaluate for any significant associations. The selection of the participants was based on random sampling of the general population and therefore the results obtained from nationally representative samples can be extrapolated to the general population. As the data collection was performed on all days of the week throughout the year in NHANES, the potential for selection bias is low [49, 50]. Moreover, the present study is one of the biggest to investigate derived dietary patterns in relation to relatively comprehensive parameters of glucose and insulin homeostasis in an American population, over time.

The present study has some limitations. Its cross-sectional nature does not allow inferences about causality. Data on glycaemic index and glycaemic load were unavailable, and accordingly, their possible effect could not be explored. In the current study, a one-day 24 h food record was used, which may not capture long-term diet. Moreover, there is a possibility of over- and underreporting food intake. Analyses in this paper are based on data collected over a 10-year period. It is likely that the diet of American people changed over this period or subsequently, which may to some extent affect the generalizability of the findings.

While the current study findings reinforce the importance of balanced diet, the uncovered links between some of the nutrients and glucose and insulin homeostasis may represent novel metabolic pathways and provide basis for further research. This raises the possibility that glucose and insulin homeostasis could be improved by changes in lifestyle, particularly by diet.

## Conclusions

This study suggests that the dietary pattern enriched with CHO, SFA, total fat and, PUFA, protein, MUFA as well as high-cholesterol-load foods is associated with impaired glucose tolerance. However, the healthy pattern which is high in vitamins, minerals and fiber appear to have favourable effects on insulin sensitivity and glucose tolerance. The principal implications of the study highlight the importance of dietary patterns in the development and prevention of dysglycaemia and impaired insulin homeostasis.
